# Insights into early recovery from Long COVID—results from the German DigiHero Cohort

**DOI:** 10.1038/s41598-024-59122-3

**Published:** 2024-04-13

**Authors:** Sophie Diexer, Bianca Klee, Cornelia Gottschick, Anja Broda, Oliver Purschke, Mascha Binder, Michael Gekle, Matthias Girndt, Jessica I. Hoell, Irene Moor, Daniel Sedding, Jonas Rosendahl, Rafael Mikolajczyk

**Affiliations:** 1 Institute for Medical Epidemiology, Biometrics and Informatics (IMEBI), Interdisciplinary Centre for Health Sciences, Medical Faculty of the Martin Luther University Halle-Wittenberg, Magdeburger Str. 8, 06112 Halle (Saale), Germany; 2 Department of Internal Medicine IV, Oncology/Haematology, Martin Luther University Halle-Wittenberg, Ernst-Grube-Str. 40, 06120 Halle (Saale), Germany; 3 Division of Medical Oncology, University Hospital Basel, Basel, Switzerland, Petersgraben 4, 4031 Basel, Switzerland; 4 Julius-Bernstein-Institute of Physiology, Medical Faculty of the Martin Luther University Halle-Wittenberg, Magdeburger Str. 6, 06110 Halle (Saale), Germany; 5 Department of Internal Medicine II, Martin Luther University Halle-Wittenberg, Ernst-Grube-Str. 40, 06120 Halle (Saale), Germany; 6 Paediatric Haematology and Oncology, Martin Luther University Halle-Wittenberg, Ernst-Grube-Str. 40, 06120 Halle (Saale), Germany; 7 Institute for Medical Sociology, Martin Luther University Halle-Wittenberg, Magdeburger Str. 8, 06112 Halle (Saale), Germany; 8 Mid-German Heart Centre, Department of Cardiology and Intensive Care Medicine, University Hospital, Martin Luther University Halle-Wittenberg, Ernst-Grube-Str. 40, 06120 Halle (Saale), Germany; 9 Department of Internal Medicine I, Martin Luther University Halle-Wittenberg, Ernst-Grube-Str. 40, 06120 Halle (Saale), Germany

**Keywords:** SARS-CoV-2, Long COVID, COVID-19, Post COVID Condition, Epidemiology, Prognosis, Infectious diseases

## Abstract

65 million people worldwide are estimated to suffer from long-term symptoms after their SARS-CoV-2 infection (Long COVID). However, there is still little information about the early recovery among those who initially developed Long COVID, i.e. had symptoms 4–12 weeks after infection but no symptoms after 12 weeks. We aimed to identify associated factors with this early recovery. We used data from SARS-CoV-2-infected individuals from the DigiHero study. Participants provided information about their SARS-CoV-2 infections and symptoms at the time of infection, 4–12 weeks, and more than 12 weeks post-infection. We performed multivariable logistic regression to identify factors associated with early recovery from Long COVID and principal component analysis (PCA) to identify groups among symptoms. 5098 participants reported symptoms at 4–12 weeks after their SARS-CoV-2 infection, of which 2441 (48%) reported no symptoms after 12 weeks. Men, younger participants, individuals with mild course of acute infection, individuals infected with the Omicron variant, and individuals who did not seek medical care in the 4–12 week period after infection had a higher chance of early recovery. In the PCA, we identified four distinct symptom groups. Our results indicate differential risk of continuing symptoms among individuals who developed Long COVID. The identified risk factors are similar to those for the development of Long COVID, so people with these characteristics are at higher risk not only for developing Long COVID, but also for longer persistence of symptoms. Those who sought medical help were also more likely to have persistent symptoms.

## Introduction

Based on conservative estimates, 65 million people worldwide suffer from long-term symptoms after their SARS-CoV-2 infection^1^. These persistent symptoms are commonly referred to as Long COVID, but there are several different terms and definitions. The World Health Organization (WHO) refers to it as “post COVID-19 condition” and defines it as symptoms persisting in individuals with a history of probable or confirmed SARS-CoV-2 infection that cannot be explained by an alternative diagnosis. For the definition to be fulfilled, these symptoms should be present three months after infection and last for at least two months^2^. The UK National Institute for Health and Care Excellence (NICE) guideline suggests a distinction between symptoms that are present between 4 and 12 weeks after infection (ongoing symptomatic COVID-19) and symptoms that persist beyond 12 weeks (post-acute COVID-19 syndrome). The term “Long COVID” is meant to include both^3^.

Long COVID comprises a wide range of symptoms. The most common symptoms include fatigue, headache, shortness of breath, muscle weakness and joint pain^4–6^. Furthermore, individuals suffering from Long COVID symptoms report worse health-related quality of life^7^. These symptoms can vary in severity and duration. Some studies have reported that symptoms persist for 24 months after infection and investigated factors associated with the recovery of symptoms^8–10^. One study showed that younger, male participants without pre-existing depression, anxiety, or cardiovascular disease were more likely to experience improvement of long-term dyspnea^11^. However, there is limited knowledge about the recovery in individuals who initially develop Long COVID symptoms and recover at an early stage.

In this study, we aimed to identify factors associated with the early recovery from Long COVID (i.e. no symptoms 12 weeks after SARS-CoV-2 infection among those who had symptoms 4–12 weeks after infection). Furthermore, we wanted to identify symptom groups present at 4–12 weeks after infection and how those are associated with early recovery.

## Methods

### Study design

The sample used in this study is part of the population-based prospective cohort study for digital health research in Germany (DigiHero, DRKS Registration-ID: DRKS00025600). The questionnaire and design of the study was described elsewhere^12^. In brief, DigiHero started in the city of Halle (Saxony-Anhalt, Germany) in January 2021 and was later extended to other federal states in Germany. Participants' addresses were taken from population registers and invitations were sent by post. After an online registration, participants received a baseline questionnaire with questions regarding socio-demographic characteristics. The current analysis is based on 48,826 participants, of which 17,008 reported at least one infection, recruited until June 15, 2022.

### Questionnaire and measures

In the baseline questionnaire, participants were asked several sociodemographic questions, including their month of birth, sex, country of birth, and education. Education was categorized into three categories (low, medium, high) based on the International Standard Classification of Education (ISCED-97)^13^. If either the participant or one of their parents was not born in Germany, we considered this as having a migration background.

Furthermore, we repeatedly asked participants if they ever had a SARS-CoV-2 infection and those who answered “yes” were subsequently invited to a dedicated questionnaire. In the questionnaire on SARS-CoV-2 infections, we asked the participants about their infection and vaccination dates. In addition, we asked whether they had symptoms and visited a doctor at the time of infection, 4–12 weeks after infection, and 12 or more weeks after infection (“Yes” and “No”). If participants reported that they had any symptoms at the specific time windows, they were asked to rate the severity of 24 different symptoms on a 6-point Likert scale from “not at all” to “very severe” and an additional option “I don’t know” (the last option was treated as a missing value in the analyzes). We categorized this into “presence of symptom” if any of the options apart from “not at all” was selected. Furthermore, participants were asked to rate their course of the acute infection (“no symptoms”, “mild”, “moderate, “severe”, and “very severe”). The last two categories were combined (“severe/very severe”). The SARS-CoV-2 variants were classified based on the reported infection date and periods of dominance of specific variants from official surveillance in Germany^14^. We classified participants as having Long COVID if they reported having symptoms 4–12 weeks after infection. Early recovery was classified if they did not report symptoms anymore for the period 12 or more weeks after infection.

For this analysis, we considered only the first infection per participant. In addition, we only included participants for whom the difference between the date of infection and the completion of the survey was more than 12 weeks, so that they could report symptoms for this period. This definition includes 11,333 participants.

### Statistical analysis

Descriptive analysis is presented using frequencies and percentages. Backward stepwise logistic regression based on the Akaike Information Criterion was used to identify possible factors associated with the early symptom recovery. The ten variables selected for inclusion in the regression analysis included the available sociodemographic factors and factors associated with the infection (sex, age, education, migration background, federal state, living in a city, self-assessed course of acute infection, virus variant combined with information on the number of previous vaccinations, whether the participant visited a doctor 4–12 weeks after infection, and an interaction term between age and sex). The variables found in the final model were used as adjustments in additional models to determine which individual symptoms present at 4–12 weeks after infection are associated with the early recovery from Long COVID.

Principal components analysis (PCA) was conducted on all symptoms for the time window 4–12 weeks after infection using the symptom scale as metric variable to identify symptom groups. To assist interpretation of the results promax rotation was used, this oblique rotation allows the factors to be intercorrelated^15^. We selected four components for the main analysis, using the scree plot (Fig. S1). To determine if a specific symptom should be included in a symptom group, a score of at least 0.40 on the primary loadings of items after rotation was used as a cutoff. The component scores were used as independent variables in a logistic regression to determine the association between the symptom groups and symptom recovery. The model was adjusted for the variables previously found to be associated with Long COVID recovery in the stepwise logistic regression.

Additionally we performed a sensitivity analysis, with a more conservative definition of Long COVID. A participant had to report at least one symptom as “moderate” to be defined as a Long COVID case and subsequently, persistence was defined only if a having long term symptoms at the time window 4–12 after infection, as well as the time window 12 weeks or more.

We report 95% confidence intervals (CI) for all analyses. All analyses were performed in R (Version 4.2.0)^16^.

### Ethical approval

The Ethics Committee of the Martin Luther University Halle-Wittenberg (2020-076) approved the study.

### Informed consent

The study was conducted following the Helsinki Declaration and informed consent was obtained from all individual participants included in the study.

## Results

### Characteristics of participants

In total, 5098 (45%) of 11,333 infected individuals reported symptoms for the time window 4–12 weeks after infection, of whom 2441 (48%) reported no symptoms for the time window after 12 weeks. The majority of the analyzed sample were female, with high education, and had no migration background (Table 1). The mean age was 46 (standard deviation = 14). Around 45% of the participants were infected during the Omicron SARS-CoV-2 period. Almost 50% of the participants classified their course of acute infection as “moderate”. Of the 5098 individuals, only 181 (4%) were hospitalized during acute infection.

**Table 1:** Characteristics of participants who reported SARS-CoV-2 infection and symptoms in the time window 4–12 weeks after infection.

		Overall	Not Recovered^a^	Recovered^a^
		N = 5098	N = 2657	N = 2441
Sex	Male	1432 (28.1%)	695 (26.2%)	737 (30.2%)
	Female	3655 (71.7%)	1956 (73.6%)	1699 (69.6%)
	Diverse	2 (0.0%)	1 (0.0%)	1 (0.0%)
	NA	9 (0.2%)	5 (0.2%)	4 (0.2%)
Age	18–29	597 (11.7%)	281 (10.6%)	316 (12.9%)
	30–39	1005 (19.7%)	429 (16.1%)	576 (23.6%)
	40–49	1068 (20.9%)	553 (20.8%)	515 (21.1%)
	50–59	1264 (24.8%)	734 (27.6%)	530 (21.7%)
	60–69	624 (12.2%)	360 (13.5%)	264 (10.8%)
	70 +	158 (3.1%)	89 (3.3%)	69 (2.8%)
	NA	382 (7.5%)	211 (7.9%)	171 (7.0%)
Migration Background	No	4204 (82.5%)	2168 (81.6%)	2036 (83.4%)
	Yes	854 (16.8%)	462 (17.4%)	392 (16.1%)
	Not specified/Unknown	40 (0.8%)	27 (1.0%)	13 (0.5%)
Federal State^b^	Saxony-Anhalt	2089 (41.0%)	1058 (39.8%)	1031 (42.2%)
	Baden-Württemberg	58 (1.1%)	31 (1.2%)	27 (1.1%)
	Bavaria	565 (11.1%)	257 (9.7%)	308 (12.6%)
	Berlin	69 (1.4%)	37 (1.4%)	32 (1.3%)
	Brandenburg	349 (6.8%)	184 (6.9%)	165 (6.8%)
	Hamburg	58 (1.1%)	25 (0.9%)	33 (1.4%)
	Rhineland-Palatinate	355 (7.0%)	185 (7.0%)	170 (7.0%)
	Saxony	1461 (28.7%)	831 (31.3%)	630 (25.8%)
	Other	14 (0.3%)	9 (0.3%)	5 (0.2%)
	NA	80 (1.6%)	40 (1.5%)	40 (1.6%)
Living in a city with 500.000 inhabitants	No	4521 (88.7%)	2360 (88.8%)	2161 (88.5%)
	Yes	497 (9.7%)	257 (9.7%)	240 (9.8%)
	NA	80 (1.6%)	40 (1.5%)	40 (1.6%)
Education	Low	199 (3.9%)	95 (3.6%)	104 (4.3%)
	Medium	1776 (34.8%)	967 (36.4%)	809 (33.1%)
	High	2833 (55.6%)	1437 (54.1%)	1396 (57.2%)
	NA	290 (5.7%)	158 (5.9%)	132 (5.4%)
Number of vaccinations preceding infection	0	2238 (43.9%)	1510 (56.8%)	728 (29.8%)
	1	230 (4.5%)	119 (4.5%)	111 (4.5%)
	2	967 (19.0%)	433 (16.3%)	534 (21.9%)
	3	1653 (32.4%)	593 (22.3%)	1060 (43.4%)
	4	10 (0.2%)	2 (0.1%)	8 (0.3%)
Variant of SARS-CoV-2	Wildtype	956 (18.8%)	706 (26.6%)	250 (10.2%)
	Alpha	892 (17.5%)	627 (23.6%)	265 (10.9%)
	Delta	980 (19.2%)	489 (18.4%)	491 (20.1%)
	Omicron	2270 (44.5%)	835 (31.4%)	1435 (58.8%)
Self-assessed course of acute infection	No symptoms	69 (1.4)	27 (1.0)	42 (1.7)
	Mild	1906 (37.4%)	801 (30.1%)	1105 (45.3%)
	Moderate	2479 (48.6%)	1353 (50.9%)	1126 (46.1%)
	Severe/very severe	638 (12.5%)	472 (17.8%)	166 (6.8%)
	NA	6 (0.1%)	4 (0.2%)	2 (0.1%)
Visited a doctor 4–12 weeks after infection	Yes	1828 (35.9%)	1252 (47.1%)	576 (23.6%)
	No	3237 (63.5%)	1395 (52.5%)	1842 (75.5%)
	NA	33 (0.6%)	10 (0.4%)	23 (0.9%)

^a^Within 12 weeks after infection.

^b^DigiHero did not target an equal coverage of all regions.

NA, not available.

### Factors associated with Long COVID recovery

Of the ten variables tested in the stepwise regression, the variables included in the final model were sex, age, self-assessed course of acute infection, the variant and vaccination status, and if participants visited a doctor in the time window 4–12 weeks after their infection. Specifically, women were less likely to recover than men were (Odds Ratio (OR) 0.80, 95% CI 0.69; 0.93). Furthermore, participants between 50 and 69 years old were more likely to still report symptoms after 12 weeks compared to the reference category (18–29 years old, OR 0.73 and 0.75, 95% CI 0.58; 0.91 and 0.58; 0.98). Participants infected during the Omicron period, independent of vaccination status, were most likely to recover early compared to all other considered variants. In addition, participants were more likely to recover early (OR 2.32, 95% CI 2.01; 2.67) if they did not seek medical care 4–12 weeks after infection (Table 2).

**Table 2:** Variables associated with early recovery (during 12 weeks after infection) from Long COVID—multivariable logistic regression.

Early Recovery from Long COVID			
		OR	95% Confidence Interval
Sex	Male	Ref	
	Female	0.80	0.69; 0.93
Age	18–29	Ref	
	30–39	1.18	0.93; 1.48
	40–49	0.82	0.65; 1.03
	50–59	0.73	0.58; 0.91
	60–69	0.75	0.58; 0.98
	70 +	0.74	0.49; 1.11
Self-assessed course of acute infection	Mild	Ref	
	No Symptoms	1.13	0.64; 2.00
	Moderate	0.74	0.64; 0.85
	Severe/Very Severe	0.45	0.36; 0.57
SARS-CoV-2 variant and number of preceding vaccinations	Omicron and 3 + vaccinations	Ref	
	Omicron and 1–2 vaccinations	0.75	0.59; 0.95
	Omicron and no vaccination	0.83	0.59; 1.17
	Delta and 3 + vaccinations	0.49	0.26; 0.92
	Delta and 1–2 vaccinations	0.55	0.44; 0.67
	Delta and no vaccination	0.41	0.31; 0.54
	Alpha and 1–2 vaccinations	0.21	0.11; 0.38
	Alpha and no vaccination	0.27	0.22; 0.33
	Wildtype and no vaccinations	0.21	0.17; 0.26
Visited a doctor in the time window 4–12 weeks after infection	Yes	Ref	
	No	2.32	2.01; 2.67

N_All_ = 4316, N_Recovered_ = 2084.

OR, Odds ratio; Ref, Reference category.

In the sensitivity analysis, using a more conservative definition for Long COVID, we identified the same variables using the stepwise regression. While the overall number of participants fulfilling the more restrictive definition of Long COVID was lower, the relative estimates were similar to the estimates for the initial definition, reported in Table 2 (Table S1).

### Single symptoms associated with early recovery from Long COVID

**Figure 1:**
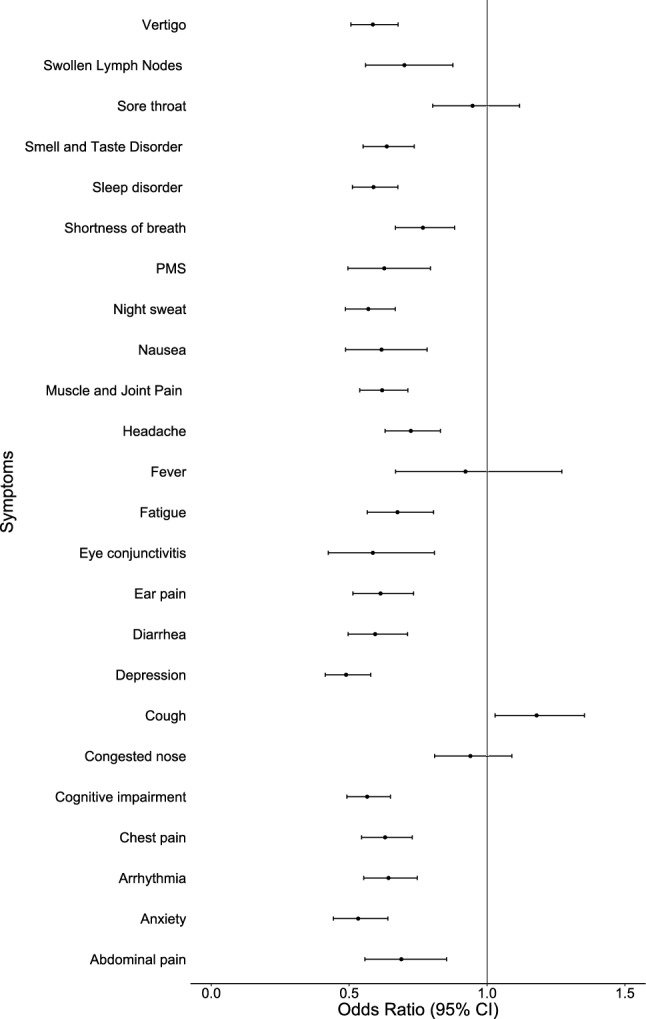
Association of symptoms present at 4–12 weeks after infection with early recovery from Long COVID, adjusted for sex, age, self-assessed course of acute infection, variant + vaccination status, and if a participant visited a doctor 4–12 weeks after infection.

We investigated the association of the presence of symptoms at 4–12 weeks after infection with the early recovery until 12 weeks. Hereby, cough was the only symptom identified that had a positive association with early recovery of symptoms (OR 1.18, 95% CI 1.03; 1.35). There was no association with early recovery for having a sore throat, fever, or congested nose. All other symptoms were associated negatively with early recovery (Fig. 1).

### Symptom groups associated with early recovery from Long COVID

We identified four distinct groups of symptoms in PCA, and four single symptoms that were not grouped (ear pain, premenstrual syndrome—PMS, swollen lymph nodes and eye conjunctivitis). The first group included diverse symptoms, described as typical symptoms associated with Long COVID like cognitive impairment and fatigue. The second group contained symptoms that could be described as symptoms of an acute infection (congested nose, sore throat, cough, and fever). The third group, termed gastrointestinal symptoms, included the symptoms abdominal pain, diarrhea, and nausea. Lastly, the fourth group was characterized by cardio-respiratory symptoms (chest pain, shortness of breath, and arrhythmia). The total variance explained by the four-factor model was 45% (Table S2).

In the logistic regression using the PCA scores, we found that symptom group 1 and 4 were negatively associated with an early recovery, while symptom group 2 was positively associated with early recovery, and symptom group 3 had no association (Table 3).

**Table 3:** Association of symptom groups using PCA scores in the time window 4–12 weeks with early recovery from Long COVID.

	Recovery of Long COVID	
	aOR^a^	95% Confidence Interval
Symptom group 1^b^	0.48	0.43; 0.55
Symptom group 2^c^	1.16	1.06; 1.28
Symptom group 3^d^	1.00	0.90; 1.10
Symptom group 4^e^	0.87	0.79; 0.96

^a^Adjusted for sex, age, course of acute infection, variant + vaccination status and if a participant visited a doctor 4–12 weeks after infection.

^b^Symptoms included: cognitive impairment, depression, fatigue, sleep disorder, anxiety, muscle and joint pain, night sweat, smell and taste disorder, vertigo, headache.

^c^Symptoms included: congested nose, sore throat, cough, fever.

^d^Symptoms included: abdominal pain, diarrhea, nausea.

^e^Symptoms included: chest pain, shortness of breath, arrhythmia.

aOR, adjusted Odds Ratio.

In the sensitivity analysis, with a more restrictive definition of Long COVID, the four identified groups were very similar. The symptoms headache, vertigo, and smell and taste disorder were not grouped anymore, however the estimates from the logistic regression using the PCA resulted in similar associations as the model presented in Table 3 (data not shown).

### Recovery from specific symptoms

**Figure 2:**
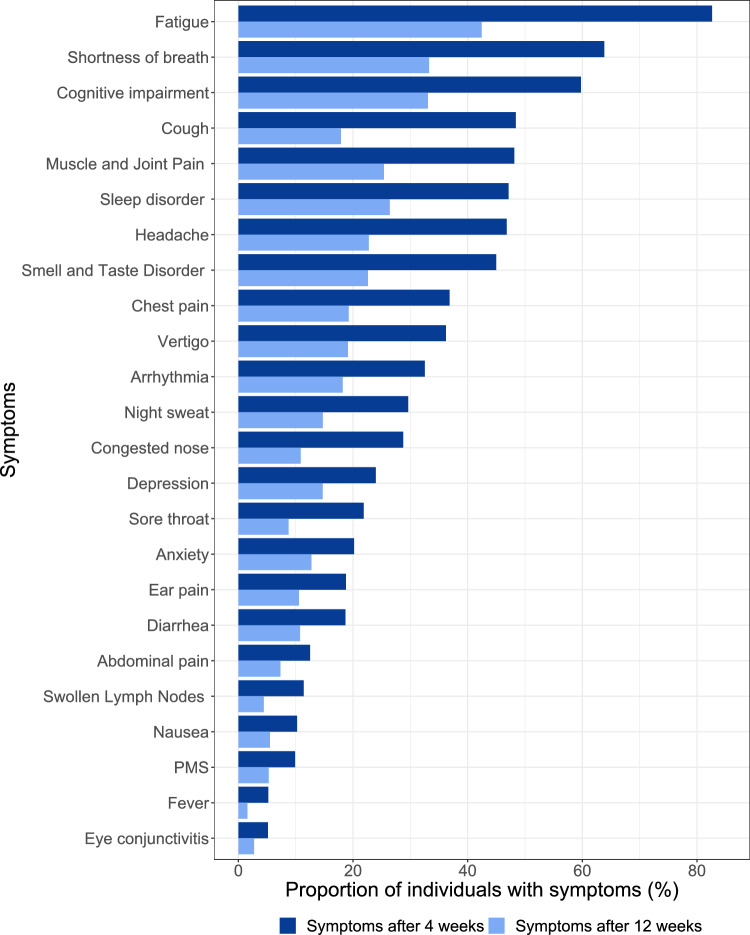
Proportion of individuals with symptoms 4 to 12 weeks and more than 12 weeks after infection.

The three most commonly reported symptoms at 4–12 weeks after infection were fatigue, shortness of breath and cognitive impairment. This did not change at the time window after 12 weeks. The greatest reductions were seen in fatigue, shortness of breath and cough (Fig. 2).

## Discussion

Using a large sample of individuals suffering from symptoms in the time window 4–12 weeks after SARS-CoV-2 infection, we studied factors associated with the early recovery from Long COVID. These factors included male sex, younger age, a milder self-assessed course of acute infection, being infected during SARS-CoV-2 Omicron dominance, and not seeking medical 4–12 weeks after infection. Additionally, having a cough at 4–12 weeks was positively associated with early recovery. Fatigue, shortness of breath, and cognitive impairment were the symptoms reported most frequently at both time windows. Furthermore, we identified four symptom groups that can be described as diverse symptoms including typical Long COVID symptoms, symptoms of an acute infection, gastrointestinal symptoms, and cardiorespiratory symptoms. The first and fourth group were both negatively associated with early recovery from Long COVID while the second group was positively associated with early recovery. This could be an indicator that there were two groups of individuals suffering from Long COVID in the initial phase. One group with symptoms, such as fatigue, that appear quickly after infection and persist later, and another group that is still dealing with lingering symptoms of an acute infection, but who will eventually recover at an early stage.

Multiple studies tried to identify Long COVID symptom clusters and patterns^17–21^. One study that looked at clusters in relation to the SARS-CoV-2 variants identified three groups of symptoms that clustered consistently across variants. These three groups included a cardiorespiratory cluster, a central neurological cluster, and a multi-organ systemic inflammatory cluster. However, overall the number of clusters differed per variant^18^. Comparable to our results one study found five clusters including gastrointestinal, airway, and cardiopulmonary clusters^19^. Another study described three clusters, where cluster one was characterized by symptoms related to pain and the other by cardiorespiratory symptoms. The third one was generally associated with less symptoms^20^. Furthermore, one study identified four distinct clusters, categorized as diverse systemic, neurocognitive, cardiorespiratory, and musculoskeletal^17^. Lastly, other research suggested three clusters where cluster 1 could be described as diverse systemic, cluster 2 included cardiorespiratory symptoms like shortness of breath, and the last one is dominated by neurological symptoms^21^. All of these studies have found a group of symptoms that include cardiorespiratory symptoms, which is similar to the symptom group 4 we identified. However, these studies used different analytic approaches to identify Long COVID symptom groups, which makes it difficult to compare the findings. Nevertheless, our findings are in line with previous studies and additionally could help in the early identification of individuals whose symptoms persist longer.

Multiple studies have identified cough as a common Long COVID symptom^4–6,21^, while we found that cough was associated with an early recovery of symptoms. However, we do not see a contradiction between these studies and our findings. Almost 20% of participants with symptoms after 12 weeks still report cough as a symptom, and while cough was associated with early symptom recovery in our study, this doesn't imply universal recovery. In our analysis, cough was grouped with symptoms such as sore throat, whereas a separate group encompassed more severe respiratory symptoms like shortness of breath, which was linked to prolonged symptom persistence. This leads us to the hypothesis that distinct groups of individuals exist, with cough potentially manifesting as either a chronic symptom or a lingering remnant of acute infection.

Most previous studies focused on identifying risk factor in regards to developing Long COVID, in contrast, there is limited information on early recovery from Long COVID. One study found that male sex is associated with recovery^22^, while another study found an association of recovery and COVID-19 severity^23^. This is in line with our findings. Several risk factors for Long COVID have been identified including female sex, younger age, smoking, a high Body-Mass-Index, and comorbidities^21,24^, and it is likely that risk factors for Long COVID also influence the symptom recovery. However, a recent study in Germany found that men were less likely to recover from cognitive deficits^25^. This is contrary to our finding that men are more likely to recover. Future studies should investigate if individual symptom recovery differs by sex. Furthermore, several studies investigated the influence of different SARS-CoV-2 variants on Long COVID risk and showed a strong risk reduction in individuals infected with Omicron SARS-CoV-2^12,17,26–28^. These findings are consistent with our results which show that having been infected during the Omicron dominance is associated with an early recovery from Long COVID. Nevertheless, more research is needed to understand which factors influence the (early) recovery of Long COVID.

We found that individuals suffering from symptoms who visited a doctor 4–12 weeks after their SARS-CoV-2 infection were less likely to recover early. A possible explanation could be that the symptoms of individuals who do not seek medical care are less severe and these individuals will then eventually recover fully. Another explanation could be that patients are already concerned about their symptoms at an early time point and therefore want to consult a general practitioner. A study identified that the “wait-and-see approach” was a common non-pharmacological intervention of German general practitioners^29^. This approach is also recommended by the German S1 guideline “Long/ Post-COVID”, in case of clinical stability of symptoms after a basic diagnosis^30^. Furthermore, a study observed the importance for patients of being believed and listened to, and at the same time that it was difficult to find a general practitioner who believed their symptoms were real^31^. Furthermore, patients participating in a German study reported that their general practitioner did not take their Long COVID symptoms seriously^32^. This could lead to an overall disappointment and mistrust. Notably, a general lack of knowledge about Long COVID was identified among healthcare professionals^33^. We believe that clinicians' understanding of Long COVID needs to be improved and that special attention should be given to individuals who seek help early. Furthermore, more research regarding Long COVID diagnosis and treatment is needed to help clinicians. Particular emphasis should be placed on the importance of early intervention for individuals experiencing persistent symptoms following SARS-CoV-2 infection. Prompt identification and management of Long COVID can mitigate the impact on patients' quality of life and long-term health outcomes.

The strength of our study is the large sample systematically recruited from the population. In contrast to studies following patients after hospital stay due to COVID-19, our sample includes mainly participants who did not require hospital treatment. Nevertheless, there are also limitations of this study. All of the information is based on retrospective self-reports, which may introduce recall bias. This could lead to an overestimation of the proportion of people suffering from Long COVID. However, we were able to show that the results were similar for a more restrictive definition of Long COVID. Additionally, we did not use an official classification for the course of acute infection, which could bias the results. Self-reporting could also lead to misclassification of infections, vaccinations and variants. In addition, we do not have information on why participants visited a doctor and what help, if any, was received. This would provide valuable insights into the care individuals receive at an early stage and their satisfaction with that care. In addition, the results might be limited to countries, like Germany, where healthcare is widely available to everyone. As the study is set in Germany, we therefore did not consider that there might be limiting factors in receiving appropriate healthcare that could negatively affect the recovery of symptoms. Furthermore, other known risk factors of Long COVID, like smoking status and comorbidities could not be taken into account, as this information was not available for DigiHero participants yet. This could lead to biased results and especially other comorbidities could also have an impact on the symptom groups. We also could not include an adequate control group with individuals not infected with SARS-CoV-2 to identify if the symptoms are unique to infected individuals. While our study offers valuable insights into Long COVID, it's essential to interpret the findings within the context of these limitations and consider avenues for future research to address these gaps comprehensively.

In summary, we identified factors and symptoms associated with the early recovery from Long COVID. There are indications that there are distinct groups of people suffering from Long COVID, those who still report lingering symptoms of an acute infection but who will recover early and the others whose symptoms will persist longer. Having sought medical help for COVID symptoms was an indicator for a higher risk of persistence.

### Supplementary Information

Supplementary Information.

## Data Availability

The anonymized data reported in this study can be obtained from the corresponding author upon request. The dataset includes individual data and an additional data dictionary will be provided. The beginning of data availability starts with the date of publication and the authors will support any requests in the three following years. Data requests should include a proposal for the planned analyses. Decisions will be made according to data use by the access committee of the DigiHero study, and data transfer will require a signed data access agreement.
