# Supplementary material for: Insights into early recovery from Long COVID—results from the German DigiHero Cohort
Source: Sci Rep. 2024 Apr 13;14:8569. doi: 10.1038/s41598-024-59122-3 (PMC11015032; doi:10.1038/s41598-024-59122-3)
Supplement: Supplementary file 1 — Supplementary Information. [file 41598_2024_59122_MOESM1_ESM.docx]

## Supplements

### Tables

| Table S1 – Variables associated with early recovery from Long COVID based on a restrictive classification of Long COVID. Recovery from Long COVID | | | |
| --- | --- | --- | --- |
|  |  | OR^a^ | 95% Confidence Interval |
| Sex | Male | Ref. |  |
|  | Female | 0.71 | 0.60; 0.84 |
| Age | 18-29 | Ref. |  |
|  | 30-39 | 1.19 | 0.93; 1.54 |
|  | 40-49 | 0.92 | 0.72; 1.18 |
|  | 50-59 | 0.83 | 0.65;1.06 |
|  | 60-69 | 0.78 | 0.59; 1.03 |
|  | 70+ | 1.03 | 0.65; 1.61 |
| Self-assessed course of acute infection | Mild | Ref. |  |
|  | No Symptoms | 0.94 | 0.50; 1.77 |
|  | Moderate | 0.72 | 0.62; 0.84 |
|  | Severe/Very Severe | 0.40 | 0.32; 0.51 |
|  |  |  |  |
| SARS-CoV-2 variant and number of preceding vaccinations | Omicron and 3+ vaccinations | Ref. |  |
|  | Omicron and 1-2 vaccinations | 0.92 | 0.71; 1.20 |
|  | Omicron and no vaccinations | 0.82 | 0.56; 1.22 |
|  | Delta and 3+ vaccinations | 0.60 | 0.30; 1.17 |
|  | Delta and 1-2 vaccinations | 0.68 | 0.54; 0.87 |
|  | Delta and no vaccinations | 0.45 | 0.33; 0.62 |
|  | Alpha and 1-2 vaccinations | 0.39 | 0.22; 0.70 |
|  | Alpha and no vaccinations | 0.32 | 0.26; 0.41 |
|  | Wildtype and no vaccinations | 0.29 | 0.24; 0.36 |
| Visited a doctor in the time window 4–12 weeks after infection | Yes | Ref. |  |
|  | No | 2.49 | 2.15; 2.88 |
| N_All_ = 3720, N_Recovered_ = 2049 | | | |
| OR, Odds ratio; Ref, Reference category | | | |
|  | | | |

Table S2: Promax-rotated factor loadings of the symptoms

| Symptoms | 1 | 2 | 3 | 4 |
| --- | --- | --- | --- | --- |
| Cognitive impairment | **0.82** | -0.15 | -0.13 | 0.03 |
| Depression | **0.79** | -0.17 | 0.07 | -0.13 |
| Fatigue | **0.73** | 0.02 | -0.18 | 0.15 |
| Sleep disorder | **0.72** | 0.03 | -0.05 | 0.06 |
| Anxiety | **0.66** | -0.20 | 0.16 | 0.04 |
| Muscle and joint pain | **0.54** | 0.17 | -0.05 | 0.12 |
| Night sweat | **0.50** | 0.09 | 0.03 | 0.06 |
| Smell and taste disorder | **0.43** | -0.02 | -0.14 | -0.32 |
| Vertigo | **0.41** | -0.05 | 0.17 | 0.28 |
| Headache | **0.41** | 0.35 | -0.02 | 0.04 |
| Ear pain | 0.31 | 0.28 | 0.05 | -0.14 |
| PMS | 0.24 | 0.03 | 0.22 | -0.15 |
| Congested nose | -0.12 | **0.81** | -0.13 | 0.03 |
| Sore throat | -0.10 | **0.77** | 0.05 | -0.14 |
| Cough | -0.22 | **0.69** | 0.22 | -0.15 |
| Fever | 0.05 | **0.41** | 0.00 | -0.13 |
| Swollen lymph nodes | 0.10 | 0.39 | 0.04 | 0.03 |
| Eye conjunctivitis | 0.04 | 0.25 | -0.10 | 0.26 |
| Abdominal pain | -0.09 | 0.00 | **0.87** | -0.04 |
| Diarrhea | -0.03 | 0.00 | **0.85** | 0.05 |
| Nausea | -0.06 | 0.00 | **0.80** | 0.02 |
| Chest pain | 0.05 | 0.01 | 0.07 | **0.77** |
| Shortness of breath | 0.09 | 0.11 | -0.11 | **0.72** |
| Arrhythmia | 0.21 | -0.13 | 0.14 | **0.59** |
|  |  |  |  |  |
| Eigenvalue | 4.06 | 2.36 | 2.36 | 2.02 |
| Variance explained | 17% | 10% | 10% | 8% |
| Cumulative | 17% | 27% | 37% | 45% |
| Factor loadings with an absolute value greater than or equal to 0.40 are shown in bold | | | | |

### Figures


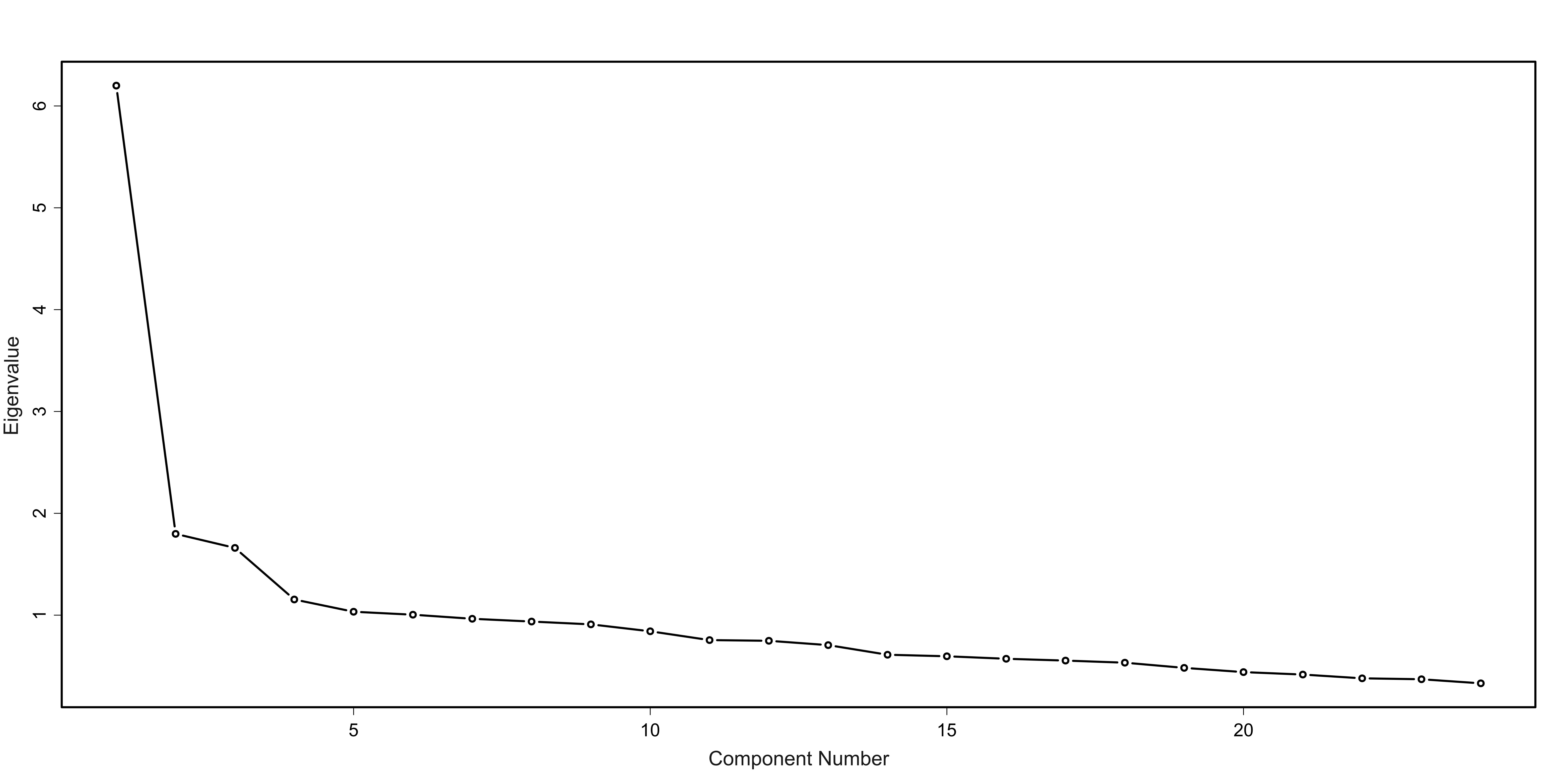


Fig. S1: Principal Component Analysis – Scree plot
